# Ultrahigh-Throughput Improvement and Discovery of Enzymes Using Droplet-Based Microfluidic Screening

**DOI:** 10.3390/mi8040128

**Published:** 2017-04-18

**Authors:** Alexis Autour, Michael Ryckelynck

**Affiliations:** Université de Strasbourg, CNRS, Architecture et Réactivité de l’ARN, UPR 9002, F-67000 Strasbourg, France; a.autour@ibmc-cnrs.unistra.fr

**Keywords:** droplet-based microfluidics, high-throughput screening, enzyme improvement, directed evolution, single-cell

## Abstract

Enzymes are extremely valuable tools for industrial, environmental, and biotechnological applications and there is a constant need for improving existing biological catalysts and for discovering new ones. Screening microbe or gene libraries is an efficient way of identifying new enzymes. In this view, droplet-based microfluidics appears to be one of the most powerful approaches as it allows inexpensive screenings in well-controlled conditions and an ultrahigh-throughput regime. This review aims to introduce the main microfluidic devices and concepts to be considered for such screening before presenting and discussing the latest successful applications of the technology for enzyme discovery.

## 1. Introduction

Enzymes constitute a fascinating class of biological polymers (proteins or nucleic acids) able to efficiently catalyze virtually any chemical reaction by placing a substrate molecule in optimal configuration and environment to stabilize the transition state, and doing so promotes its transformation (e.g., cleavage, addition, or modification) in an enantioselective manner [1]. These properties make enzymes extremely attractive for industrial applications (e.g., green chemistry, bioethanol production), bioremediation, biotechnologies, bioengineering, and synthetic biology. However, even though a plethora of natural enzymes have been identified, improvement and/or adaptation of existing molecules, or even discovery of new ones, may still be necessary for several reasons [2]. First, the wild-type molecule has naturally evolved to display an optimal activity in physicochemical conditions (e.g., crowded medium, temperature, ionic strength…) that may significantly differ from those of the application one may wish to use it for [3]. Second, the activity of interest may be a secondary weak activity of the molecule. This scenario is typically encountered with promiscuous enzymes, a set of catalysts with relaxed specificity that are able to catalyze other reactions and/or transforming other substrates than those they originally evolved for [4]. Promiscuity has been proposed to be an important evolutionary engine predisposing the cell with a basal level of activity allowing for gene speciation and rapid adaptation to environmental changes [5]. However, the prediction of such activity, as well as the identity of the residues to mutate to improve this activity, is extremely challenging. Finally, the catalyst may have been lost during the evolution as it is the case of many catalytic RNAs that one may want to resurrect, for instance, when studying the origin of life [6]. 

In any of the aforementioned cases, the catalyst can be improved, or generated *de novo*, using directed evolution, a set of laboratory methodologies allowing for rapid evolution of biological molecules (proteins or nucleic acids) by iterative rounds of (i) mutagenesis to generate mutant libraries (genetic diversity) and (ii) selection aiming at enriching the libraries in molecules with the desired properties [7,8,9,10,11,12,13,14]. Owing to the 20-letter alphabet of proteins (4-letter for nucleic acids), the size of gene libraries rapidly grows with increasing the number of residues to be mutated. Therefore, the selection process has to be able to handle such a large diversity (>10^6^ mutants). Affinity panning technologies can easily manipulate such diversity [6,15] but, whereas they are very efficient at identifying good binders, these approaches are less suited for enzyme evolution since a single turnover is often sufficient to make a gene selectable, which preclude the enrichment of molecules with elevated turnover in the library. Consequently, a high-throughput screening approach where each variant of a library needs to perform several turnovers to become selected has a better chance of successfully identifying efficient enzymes. 

In this review, we present the latest developments and use of droplet-based microfluidics for the discovery of new enzymes by ultrahigh-throughput screening of either mutant libraries or environmental samples. This is however only one of the various applications of the technology and the reader is referred to other recent reviews for a more exhaustive view of its scope of application [16,17,18,19].

## 2. (Ultra) High-Throughput Screening Strategies and Their Limitations

**Figure 1:**
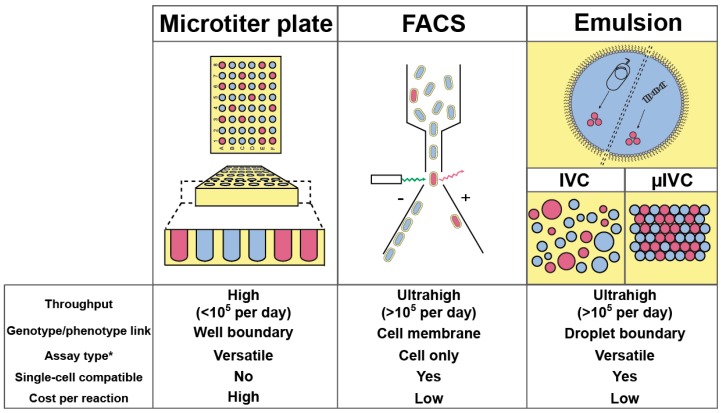
Principal (ultra)high-throughput screening strategies for enzyme improvement/discovery. The different methods (Microtiter plate, fluorescence-activated cell sorting (FACS) and emulsion) are schematized with the boundary (well, cell membrane, or oil) confining the phenotype with the genotype shaded in yellow. High-throughput (up to 10^5^ analyses par day) is distinguished from ultrahigh-throughput (more than 10^5^ analyses per day) regimes. Note that the emulsion-based approach is represented its two format: (i) in vitro compartmentalization (IVC) manipulating polydisperse emulsions and (ii) microfluidic-assisted IVC (µIVC) manipulating highly monodisperse emulsions. Compartments containing the target phenotype (red) are discriminated from those that do not possess it (blue). *Assay type can be cell-based, cell lysate-based, cell-free, or all of them (versatile).

Nowadays, most high-throughput screenings aimed at improving/discovering enzymes are performed by specialized facilities using microtiter plate (MTP) format and requiring sizeable, costly, and sophisticated colony pickers and liquid-handling robots able to perform up to 10^5^ assays per day (Figure 1) [20]. Therefore, screening a million variants with such platform would take at least 10 days. Increasing MTP well density from 96 (100–200 µL per well) to 384 (30–100 µL per well) and even 1536 (2.5–10 µL per well) wells per plate can increase analytical throughput while decreasing the cost. However, further reduction is prevented by capillarity and fast evaporation that would dominate at sub-microliter volumes. Beside MTPs, the fluorescence-activated cell sorter (FACS) is common bench-top laboratory equipment able to analyze and sort up to 10,000–15,000 cells per second, an ultrahigh-throughput regime (more than 10^5^ analyses per day) allowing a million variants to be screened in less than 2 min. However, whereas in MTP the well boundary physically confines the genotype (catalyst-coding gene) with the phenotype (reaction product), the use of the FACS requires this linkage to be performed at the level of the cell [21]. Therefore, FACS-based screenings are restricted to only two scenarios: either the produced enzyme stays in the cytoplasm and the fluorogenic substrate has to be able to cross the cell membrane while the fluorescent product should stay trapped in the cell; or the enzyme is displayed at cell surface and the fluorescent product should be captured at cell surface as well.

Emulsion-based screening represents a third strategy in which a biological (or chemical) reaction is dispersed into an oil phase to form water-in-oil (w/o) droplets, whose boundary ensures the genotype/phenotype linkage [22]. As originally conceptualized by Tawfik and Griffiths [23], in vitro compartmentalization (IVC) was limited to DNA modifying enzymes or required complex chemistry and/or selection procedure to be set up [24,25]. However, the possibility of re-emulsifying w/o droplets into water-in-oil-in-water (w/o/w) double emulsions made it possible to screen droplets using fluorogenic assays and FACS [26]. By combining the advantages of MTP and FACS, IVC allowed improving the activity of many enzymes [27,28,29,30,31,32,33,34,35,36,37,38], a list that may lengthen in the future with the development of new strategies [39]. Finally, the small droplet volume (femtoliters to picoliters) permits screening directly at the level of single cells, and even single genes. Despite the great perspectives opened by the IVC, the technology still suffered limitations challenging its flexibility and that could ultimately preclude the identification of efficient catalysts [24,25]. Indeed, high polydispersity within emulsions significantly limits IVC quantitatively, a phenomenon further amplified when a second emulsification step is used as it may lead to the co-encapsulation of several w/o droplets into a single w/o/w droplet, increasing the false positive rate. Finally, the difficulty of modifying the content of a droplet once it has been formed strongly reduces IVC flexibility. Nevertheless, all these limitations can be overcome by transposing IVC to a microfluidic format, summarized hereafter as µIVC; standing for microfluidic-assisted In Vitro Compartmentalization. 

## 3. Droplet-Based Microfluidics

Setting up a µIVC screening pipeline requires generating monodisperse water-in-oil droplets but it may also necessitate to modify their content and, at the end of the process, the enzymatic activity contained in each droplet should be quantified and used to sort them accordingly.

### 3.1. Droplet Production

**Figure 2:**
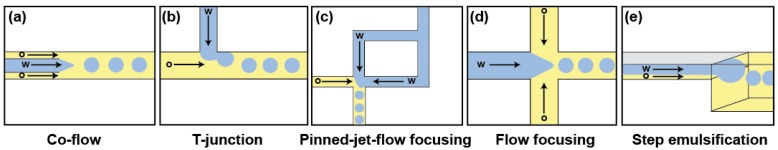
Main geometries used for droplet generation. (**a**) Droplet generation by co-flowing an aqueous phase (in blue) arriving from a capillary immersed in an oil stream (yellow). (**b**–**d**) Droplet generators with planar geometries. Channels in which oil (shaded in yellow) and aqueous phase (shaded in blue) flow are respectively labeled “o” and “w”. Arrows indicate flow directions. (**e**) Schematic of a step emulsification process.

Water-in-oil (w/o) emulsions are obtained by dispersing an aqueous phase into an immiscible carrier oil phase, usually supplemented with a surfactant molecule to stabilize the emulsion (see Section 4). However, because of the laminar flow adopted by liquids circulating within a microfluidic device, simply flowing the continuous (oil) and the dispersed (aqueous) phases together in a channel is not enough to generate droplets of controlled volume. Instead, the production of highly monodisperse emulsions (less than 5% of variation in droplet volumes) requires the use of well-defined geometries, eventually producing fL to nL droplets. Many geometries and strategies have been developed over the last years. Whereas an exhaustive introduction of these systems can be accessed elsewhere [40], the main strategies are summarized on Figure 2.

A first set of droplet generators for producing droplets of reliable and controlled size used geometries that apply a shear stress to the dispersed phase. In early 2000s, Umbanhowar et al. first introduced the co-flow strategy (Figure 2a) where the dispersed phase is released from a capillary into a carrier oil phase flowing in a parallel direction outside the capillary [41]. Nevertheless, the complexity of integrating capillaries with conventional microfabrication processes has precluded the wide use of such devices. Nowadays, the most widely used droplet generators have planar geometries consisting of intersecting channels bringing together an aqueous and an oil flow (Figure 2b–d). Among them, T-junction [42,43] is frequently used for the production of large droplets while hydrodynamic flow-focusing [44] is preferred for generating droplets of small size and/or at very high production frequency. Moreover, the pinned-jet flow focusing [45] is an attractive variation of the T-junction, allowing for producing droplets at a production frequency matching the operating frequency of a downstream module (e.g., droplet fuser or droplet sorter). 

Beside shearing-based strategies, droplets can also be produced through a sharp change in the capillary pressure as this occurs during step emulsification. On such a device (Figure 2e), the aqueous and continuous phases are first combined in a shallow region acting as a Hele–Shaw cell that prevents droplet formation by suppressing the interfacial instability. However, when the liquids reach the much deeper region of the device, the abrupt relaxation of the confinement leads to droplet formation [46,47]. Interestingly, an abrupt step is not an absolute requirement, since the effect can be obtained by using a progressive gradient of confinement [48]. Step emulsification is well suited for producing monodisperse emulsions even in extreme conditions of viscosity [46] as well as for the production of monodisperse emulsions made of small femtoliter droplets [49].

Upon droplet production, their contents start to mix because of recirculating streamlines inside the droplet [50], but faster mixing (less than 2 ms) can be achieved by circulating the droplets into a zigzag-shaped channel inducing chaotic advection into the droplet [51]. Finally, droplets can be incubated on-chip, either by storing them in dedicated chambers [52,53] and microarrays [54]; or by circulating them into various geometries of channels [55,56]. Alternatively, droplets can be collected into a variety of off-chip reservoirs (e.g., tube closed by a polydimethylsiloxane (PDMS) plug [57], a capillary [58], a Pasteur pipette [59], or a syringe [60]) and incubated for longer times.

### 3.2. Droplet Content Modification

**Figure 3:**
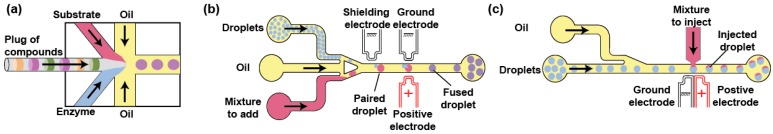
Main strategies for modifying droplet content. (**a**) Modification before droplet production. Plugs of compounds spaced by a carrier liquid (in gray) are injected into a droplet generator and combined with two other aqueous phases (containing enzyme and substrate) just prior to being emulsified. (**b**) Droplet fusion device. Schematic of an active droplet fusion device where droplets are synchronized and fused when passing between a pair of electrodes. Here, small droplets (blue) are reinjected, spaced, and synchronized with larger droplets (red) generated on-chip. (**c**) Droplet picoinjection device. Droplets are reinjected and spaced by an oil stream. When passing in between an orthogonal channel containing the fluid to inject and a pair of electrodes that destabilize droplet/liquid interface, the droplets receive a controlled volume of fluid. Arrows indicate flow directions.

A great advantage of droplet-based microfluidics is the possibility of modifying droplet composition on demand and in a controlled manner. For instance, combining different aqueous streams just upstream the droplet production nozzle makes it possible to continuously produce droplets while modulating their content by changing the composition of one of the streams. For instance, plugs of compounds (e.g., enzyme inhibitors, substrates…) can be prepared from microtiter plate [61,62] or tubes [63,64,65] and sequentially injected into the droplet generator (Figure 3a). Alternatively, compounds can be loaded onto beads prior to individualizing them into droplets and release their payload by UV illumination [66]. Droplets of variable but controlled composition can also be produced by ‘printing’ them using valve-based droplet generators [67,68]. Moreover, the concentration of the reagents added to the droplets can be varied using one of the several reported microfluidic gradient generator [69,70], a concentrator module [71], or an ultra-performance liquid chromatography [72].

Droplet content can also be modified after the droplet has been formed and stabilized by a surfactant, which is especially important when implementing an experimental workflow in which two (or more) mutually incompatible steps must be decoupled (e.g., cell growth and activity assay [73,74,75], or protein synthesis and activity assay in harsh conditions [76]) as this may occur in some µIVC procedures. A first approach consists in fusing each droplet with a second one containing the reagents to be delivered [77] (Figure 3b). Pairs of droplets can be formed and synchronized using dedicated structures such as pillar arrays [78] or by exploiting the Poiseuille parabolic profile adopted by a liquid circulating in a microfluidic channel, which causes a small droplet to circulate faster than a larger one, eventually leading the former to catch up the latter [79]. If one of the droplets is deprived of surfactant [80] or only partially stabilized [81], the pairs of droplets can be passively fused upon a decompression, likely as the result of a local depletion of surfactant molecules [82]. However, this approach may be sensitive to surfactant batch-to-batch variations. Therefore, active strategies in which an external event triggers droplet fusion are more attractive due to their robustness. Laser-assisted local warming of droplet–droplet interface was shown to efficiently promote droplet fusion [83] but is not frequently used as it can damage the biological sample. Instead, most of the active droplet fusion devices use either charged droplets [84] and more frequently electrocoalescence where an AC field is used to destabilize droplets’ interface and promote their fusion (Figure 3b) [85]. Electrodes can be directly inserted into the channel [86], built under it [79] or, more frequently, made of a solder [87] or a salt solution [88] injected in channels surrounding the channel of interest.

Finally, addition of reagents to surfactant-stabilized droplets can also be performed by the direct injection of the solution into circulating droplets [89] (Figure 3c). In a droplet picoinjector, a solution is delivered to the droplets while they pass in front a pressurized injection channel subjected to an electric field that transiently destabilizes the droplet/aqueous solution interface. Even though this module can be prone to hydrodynamic instabilities, the implementation of an upstream pressure stabilizer was found to greatly improve its reliability [90].

### 3.3. Droplet Analysis and Sorting

The last step of a µIVC screening consists in measuring the phenotype (i.e., the enzymatic activity) of each droplet and using it to trigger the specific recovery of droplets containing the corresponding genotype.

Over the past decade, a large set of analytical techniques has been adapted to droplet-based microfluidics as reviewed in [91]. These techniques include label-free methods such Raman spectroscopy and mass spectrometry, but also electrochemical detection of reaction products and, more frequently, optical detections exploiting the plethora of fluorogenic and chromogenic substrates commercially available. Light-induced fluorescence (LIF) is currently the most-used detection approach as it offers high sensitivity, makes possible to track several parameters (e.g., enzyme activity, droplet size…) in parallel by using different colors, and is compatible with high throughput measurements on droplets circulating with a short residence time in the analytical area. Indeed, since the fluorescence lifetime (ns) is much lower than the time a droplet spends in the analysis window (µs-ms), the analysis time is not a limiting factor, making it possible to analyze the fluorescence of several tens of thousands of droplets per second. Most of the LIF-based assays are performed using continuous illumination at a single measurement point giving access only to a snapshot of the reaction. However, recent work showed that, even though at a lower throughput (150 droplets per second), reaction kinetics could be accessed by combining wide field microscopy and stroboscopic illumination [92]. Moreover, despite the small optical path length of the droplets, recent studies have demonstrated the possibility of monitoring droplet absorbance [93,94], which opens exciting perspectives by expanding the range of assays achievable in droplets.

**Figure 4:**
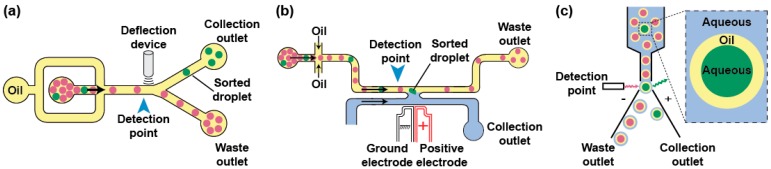
Main droplet sorting strategies. (**a**) Microfluidic droplet sorting using droplet deflection. Droplets are reinjected into a sorting device and spaced by an oil flow. Droplet content (e.g., fluorescence) is analyzed at a detection point (blue arrow) and a deflection device (e.g., pairs of electrodes, surface acoustic wave generator, piezoelectric component…) is activated accordingly to deflect the droplets of interest (green) into the collection channel. (**b**) Droplet sorting based on droplet fusion with an aqueous phase. Droplets are reinjected into the sorting device and spaced by an oil flow. Droplet content (e.g., fluorescence) is analyzed at a detection point (blue arrow) and a pair of electrodes is activated to fuse the droplet of interest with a continuous aqueous flow (blue) accordingly. (**c**) FACS-based droplet sorting. Water-in-oil-in-water droplets are carried by a sheath fluid (blue), analyzed and sorted by FACS.

Phenotypes can be used to trigger the specific recovery of the droplets of interest by deflecting them into a dedicated channel. Typical sorting devices consist of a re-injection module where the droplets are injected and spaced by an oil stream before arriving at a junction where the main channel splits into at least two exhaust channels: the “waste” channel into which the droplets flow per default and the “collection” channel the droplets of interest are deflected in (Figure 4a). As recently reviewed elsewhere [95], there is a large variety of geometries and active strategies that can be used to sort droplets including laser-induced thermophoresis [83], mechanical sorting using piezoelectric elements [96] or single-layer built-in membrane valves [97], acoustic waves [98,99], as well as electrical approaches in which droplets are charged on-chip prior to being deflected into one [84] or several channels by dedicated sets of electrodes [100]. One of the most used deflection methods in µIVC is based on dielectrophoresis (DEP). In DEP, the application of an AC field to a pair of electrodes creates a non-uniform electric field polarizing the droplet and pulling it toward the most intense part of the field, which ultimately leads to its deflection toward the “collection” channel [101]. Depending on the biological assay, integrating a fluorescence or absorbance read-out with DEP sorting produces FADS (Fluorescence-Activated Droplet Sorting) [59] and AADS (Absorbance-Activated Droplet Sorting) [94] microfluidic platforms, respectively. Recently, an optimized FADS geometry allowed sorting up to 30,000 droplets per second (30 kHz) [102]. While the continuous phase circulating in the sort channel usually consists of the fluorinated oil spacing the reinjected droplets, the use of a H-shaped geometry allows filling the collection channel with an aqueous stream so that, upon a sort signal, a droplet is both deflected and its content (a bead [103] or a gene [104,105]) is extracted into an aqueous phase (Figure 4b). 

Aside from the microfluidic droplet sorting devices, droplets can also be handled on a conventional FACS (Figure 4c), provided the continuous oil phase is exchanged for an aqueous phase. A first strategy consists of generating a double emulsion by re-emulsifying water-in-oil droplets into water-in-oil-in-water droplets carried by a sheath fluid [106]. Alternatively, droplets can be converted into hydrogel beads [107]. For instance, droplets can initially contain a polymer (e.g., agarose) kept in a liquid form and gelified only after the droplet has been formed. Upon droplet breaking, the beads form a physical boundary that confines compartment content and can be further tightened using additives such as charged polymers [108]. The beads can then be suspended into conventional sheath fluid prior to being loaded, analyzed, and sorted on a FACS machine.

## 4. Genotype/Phenotype Confinement Maintenance

The success of µIVC screening is primarily conditioned by the proper maintenance of genotype/phenotype confinement. However, this linkage can be challenged by two main scenarios: (i) an uncontrolled coalescence resulting from a lack of droplet stability and (ii) droplet–droplet exchange of reaction products, especially fluorescent dyes, leading to an uniformization of droplet phenotypes. In both cases, various strategies have been proposed to limit these adverse effects.

### 4.1. Droplet Stabilization

**Figure 5:**
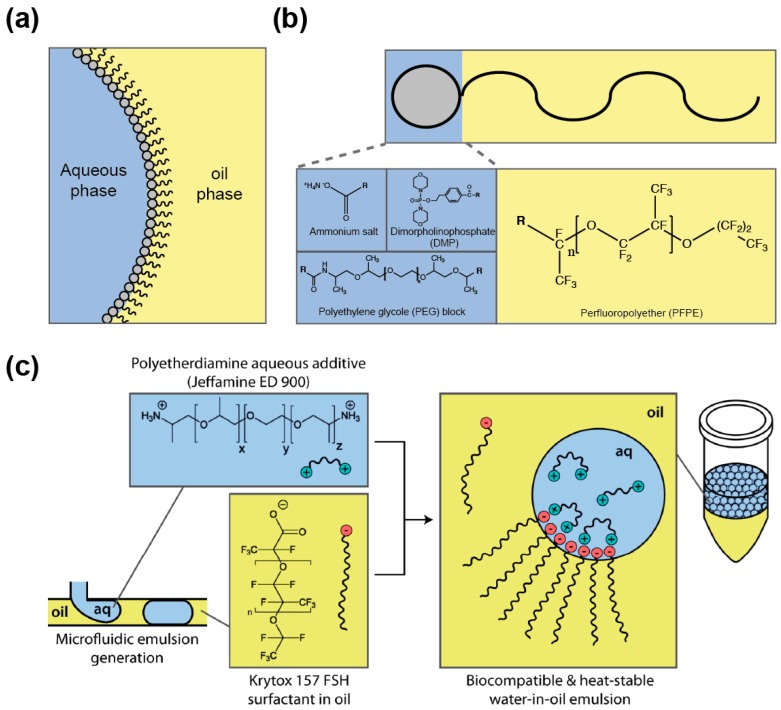
Chemical structure of fluorinated surfactants. (**a**) Surfactants are amphiphilic molecules that stabilize droplets by partitioning at water/oil interface. (**b**) Structure of the fluorinated surfactants. Perfluoropolyether (PFPE) fluorinated tail (shaded in yellow) is covalently attached to different polar head groups (shaded in blue). (**c**) Droplet stabilization by bipartite surfactant. Krytox™ 157 FSH fluorinated tail (shaded in yellow) stabilizes the droplet by partitioning at the interface and interacts with Jeffamine ED 900 polar head group (shaded in blue) by ionic interactions. Reprinted with permission [117]. Copyright 2013 American Chemical Society.

Dispersing an aqueous medium into an immiscible oil phase allows producing droplets whose interface is subjected to a high surface tension. As a consequence, droplets tend to coalesce to minimize oil/water surface area, a phenomenon that disrupts genotype/phenotype linkage. Nevertheless, droplet stability can be preserved by supplementing the oil phase with a “surface active agent” (or surfactant). Surfactants are amphiphilic molecules composed of different groups having affinity for each phase (oil and aqueous) and that are able to reduce the surface tension by partitioning at the oil/aqueous interface (Figure 5a). Once at the interface, the surfactant was proposed to prevent droplet coalescence by steric hindrance as well as by the establishment of a Marangoni flow, counteracting the oil drainage between droplets contacting each other [109]. A variety of oils and surfactants can be used as reviewed elsewhere [109,110] and, whereas IVC protocols usually employ non-fluorinated oils [111], nowadays droplet-based microfluidic approaches mainly use fluorinated carrier oils (e.g., FC40 and Novec 7500) since they both limit droplet–droplet molecular exchanges, as organic molecules are poorly soluble in these oils [112], and they are well compatible with PDMS (avoiding PDMS swelling [113] as mineral oil would [52]). In addition, fluorinated oils efficiently dissolve respiratory gases [114], a particularly interesting feature when cells need to be grown in the droplets. Most of the fluorinated surfactants are based on the perfluorinated polyether (PFPE) Krytox™ (Figure 5b) that possesses a carboxylic head group. However, the ammonium salt of the molecule has poor biocompatibility [115], a key parameter that can be efficiently assessed using cell-based [115] or in vitro gene expression [116] assays. The toxicity of Krytox™ was likely due to the nature of the counter cation used, since a recent study showed that the addition of free polyetherdiamine in the aqueous phase (Figure 5c) restores the biocompatibility of droplets stabilized by Krytox™ [117]. However, most of the surfactants used nowadays consist of PFPE tails covalently conjugated with polar heads like dimorpholinophosphate [115], polyethylene glycols [118], or polyetherdiamine [71]. Several of these block co-polymers are now commercially available (e.g., from RAN biotechnologies (Beverly, MA, USA) or Sphere Fluidics (Cambridge, UK)) which eases the access to the technology. Furthermore, since surfactant toxicity may be due to non-specific adhesion to surface, additives such as Pluronic®, can be added to the aqueous phase to limit adsorption of cells or molecules [119].

Besides its function in droplet stabilization, the surfactant may also fulfill additional roles. For instance, using a surfactant affording a polar head functionalized with a nitrilotriactetate group was used to both stabilize the droplets and capture his-tagged green fluorescent protein at their surface [120]. Similarly, gold-conjugated surfactant was used to immobilize peptides for capturing cells at the droplet surface [121]. Finally, inner droplet surface could also be functionalized using surfactant affording hydroxyl groups such as the fluorinated polyglycerols [122] or *via* bio-orthogonal click chemistry using azide surfactant [123], both approaches opening up exciting perspectives in future applications exploiting the large inner droplet surface.

### 4.2. Limiting Droplet–Droplet Exchange

Preserving the genotype/phenotype link also requires preventing information exchange between the droplets. Bulky charged genetic polymers (RNA and DNA) are unlikely to easily pass through the droplet/oil interface. However, smaller molecules like fluorescent products (phenotype) are more prone to be released from and/or exchanged between the droplets. Such a leakage can occur in two different ways: (i) partition of the compound into the oil phase or (ii) micellar transport. Whereas direct partition of the dyes into the oil phase has been described for droplets carried by hydrocarbon oils [124,125], the phenomenon is less common in fluorinated oils even though some dyes like coumarin were found to efficiently diffuse into Novec 7500 oil [126]. Molecular retention is however strongly challenged by micellar transport, a dynamic process that has been modeled [127,128] and in which the free surfactant contained into the oil self-organizes into micelles that can act as cargo, transporting molecules from one droplet to the other. Micelles formation can be limited by working at surfactant concentrations below the critical micellar concentration (CMC) at which they start to be significant, but this would also challenge droplets stability. Therefore, several strategies have been explored to limit micellar transport while working at optimal surfactant concentrations. Simple actions such as adjusting surfactant concentration and maintaining spacing in between droplets were shown to significantly decrease exchange kinetics [129]. Nevertheless, this may not always be applicable, especially when extended incubation times are required, as it is the case during most of the biological screenings, and for which droplets are incubated as dense and compact emulsions. 

**Figure 6:**
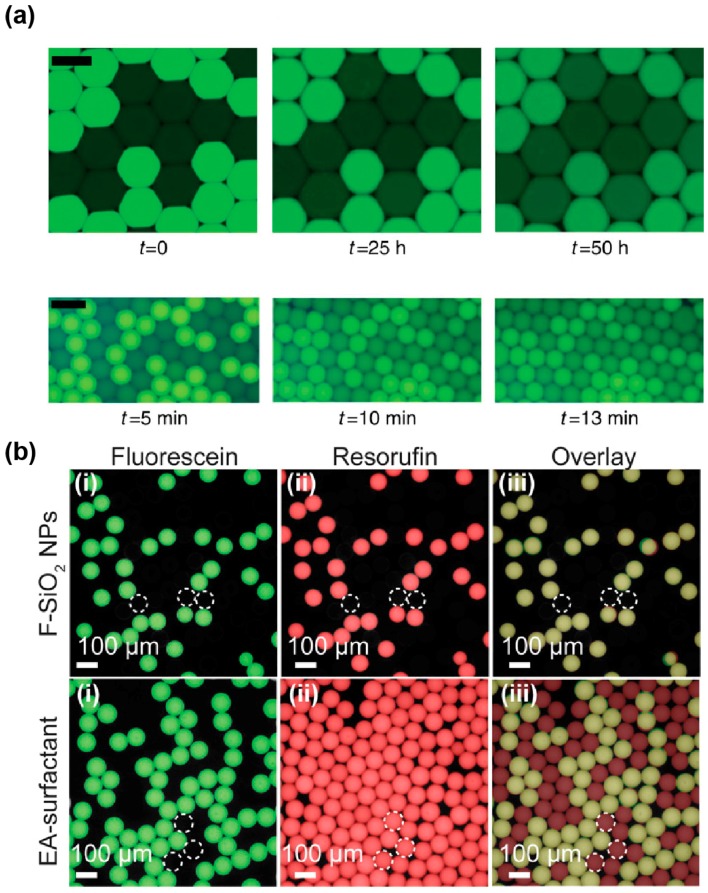
Retention of different fluorescent dyes into droplets. (**a**) Retention of fluorescent dyes into droplets stabilized by a surfactant. Whereas fluorescein has an extended retention time (top panel) in surfactant-stabilized droplets, the rhodamine 6G is rapidly exchanged (bottom panel). Adapted from [129] under a Creative Commons License. (**b**) Retention of fluorescent dyes into droplets stabilized by a nanoparticle. Whereas both fluorescein and resorufin are efficiently confined into nanoparticle-stabilized droplets (top panel), the latter is rapidly exchanged between surfactant-stabilized droplets (bottom panel). Adapted with permission from [135]. Copyright 2014 American Chemical Society.

The degree to which a dye is loaded into micelles differs from one molecule to other. Indeed, while some dyes, like fluorescein (Figure 6a), have a retention time in droplets of several days, others like rhodamine 6G [129], coumarin [126], and resorufin [127] tend to have very fast leakage kinetics with retention times in the order of seconds to minutes. Several works have correlated the propensity of a dye to be exchanged by micellar transport with its distribution coefficient (LogD) [126,130], suggesting that molecules of higher hydrophobicity are more likely to be efficiently loaded into micelles. Therefore, increasing the hydrophilicity of the dye by adding polar groups was expected to reduce micellar transport of the molecule. This hypothesis was first validated by the observation that substituting a coumarin with a sulfonate group increased its retention time in the droplets from a few minutes to hours and even days [126,131]. Addition of hydrocarbon (dodecyl) groups was also found to significantly increase the retention of resorufin by a mechanism that still needs to be deciphered [132]. Beside the chemical modification of the fluorophore, Janiesch et al. showed that tailoring buffer composition, as well as surfactant geometry and concentration, to the properties of the dye may also be used to modulate its retention [130]. Reaction mixtures can also be supplemented with additives such as sugars [133] and bovine serum albumin (BSA, [134]) that were both found to significantly limit fluorescent dye leakage. While the exact mechanism of action underlying sugars is unclear, BSA was suggested to act by increasing the solubility of the fluorophore into the droplets [127].

Finally, a new solution to both stabilize droplets and efficiently prevent droplet–droplet exchanges may come from the use of nanoparticules (NPs) instead of conventional surfactants. Indeed, a recent report showed that fluorinated silica NPs efficiently stabilize droplets carried in a surfactant-free oil phase, while abolishing resorufin leakage (Figure 6b) [135] and being biocompatible [136]. For µIVC screening applications, these attractive properties might however be balanced by the rather high stability of the interface. Indeed, the authors pointed out that NPs were likely to irreversibly adsorb at the interface, raising questions about the possibility of modifying droplet content after they have been formed, a limitation shared with IVC. In addition, the possibility of recovering the content from droplets of interest still needs to be addressed.

## 5. Discovery and Improvement of Biological Catalysts

Combining several of the microfluidic devices introduced above, together with the use of proper reagents, allowed devising µIVC screening workflows for the isolation of efficient catalysts, either from mutant libraries by directed evolution of natural or synthetic molecules, or through the bioprospecting of new activities from environmental samples. In any case, the low cost and the ultrahigh-throughput of the µIVC are two key factors enabling rapid and efficient screening of large libraries for rare events that would be extremely difficult to identify by lower throughput approaches such as MTPs.

### 5.1. Cell-Based Directed Evolution

The first directed evolution performed with µIVC was achieved by Agresti et al. who developed an integrated microfluidic platform composed of a droplet generator directly connected to a FADS module by a delay line, which was used to improve the catalytic properties of the horseradish peroxidase (HRP) [137]. HRP mutant gene libraries were prepared by error-prone PCR prior to being expressed in yeast as surface-displayed proteins. Since cell encapsulation follows a Poisson distribution [115,138], adjusting the dilution of cell suspension allowed encapsulating single cells in the presence of a fluorogenic substrate of the enzyme, therefore confining a single genotype with its phenotype. Upon short on-chip incubation, droplets presenting the highest fluorescence signal were sorted and the cells were recovered. Performing a few rounds of screening identified improved variants of the already efficient HRP that were able to catalyze the reaction (10 times higher catalytic constant *k_cat_*) with a near diffusion-limited efficiency. A second major outcome of this work was the demonstration of the significant saving of time (1000 times faster) and cost (1 million times cheaper) allowed by such µIVC workflow with respect to a conventional robotic-based screening. 

**Figure 7:**
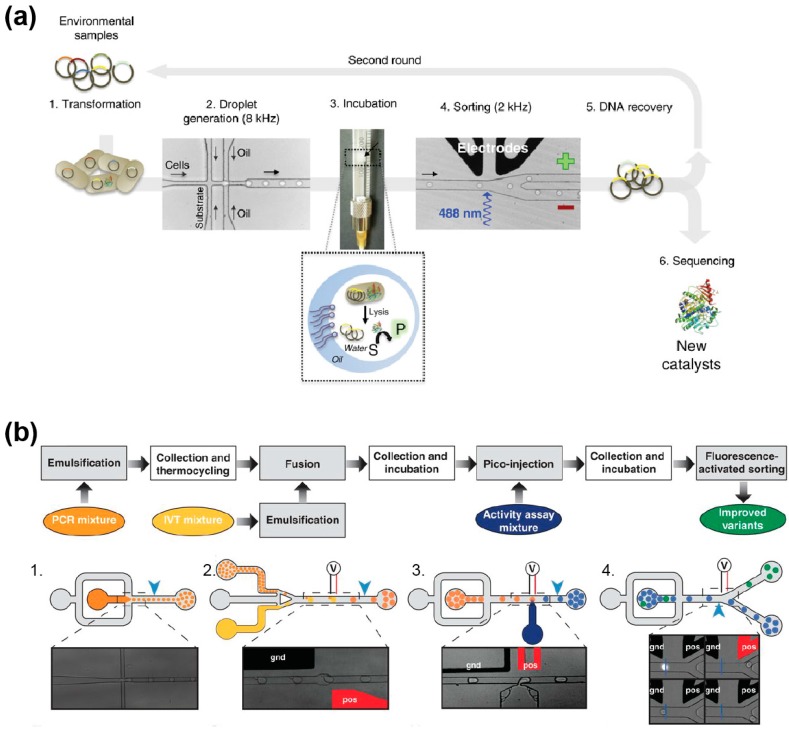
Microfluidic workflow for directed evolution of biological catalysts. (**a**) Cell-based µIVC screening workflow. Bacteria were transformed with a metagenomic library from an environmental sample (1) and individualized into droplets together with a fluorogenic substrate and a lysis agent (2). Upon collection and incubation in a syringe (3), the emulsion was reinjected into a microfluidic droplet sorter (4), the positive droplets were sorted and the DNA recovered (5). The variants were either analyzed or used for a new round of screening (6). Adapted from [147] under a Creative Commons License. (**b**) Cell-free µIVC screening workflow. Genes of a library were individualized into PCR droplets (1) and, upon DNA amplification by thermocycling, each droplet was synchronized and fused with a droplet containing an in vitro transcription mixture (2). Once the transcription occurred, a substrate solution was delivered to each droplet by picoinjection (3) prior to a short incubation followed by droplet fluorescence analysis and sort (4). Adapted from [148] under a Creative Commons License.

While yeast display allows protein synthesis in a eukaryotic context, gene libraries can also be expressed in prokaryotes, especially in the bacterium *Escherichia coli*, with the protein of interest accumulating either in the periplasm or in the cytoplasm. The former allows the substrate to directly access the enzyme as shown by a model experiment in which *Escherichia coli* cells expressing *Bacillus subtilis* CotA laccase were subjected to a µIVC procedure to profile the activity of a mutant library [73]. Nevertheless, most of the evolutions performed so far used cytoplasmic expression of the protein, requiring the cell to be lysed into the droplet to release the enzyme [60,94,105,108,139,140,141]. In these approaches (Figure 7a), the cell suspension is first combined on-chip with an aqueous stream containing a lysis agent (usually a detergent, but the combined use of lysozyme with an electric field [141] and heat-lysis [139] were also demonstrated) and a fluorogenic substrate prior to generating water-in-oil droplets [60,142]. Droplets are then incubated either on-chip [140] or off-chip [60,94,105,108,139], prior to being analyzed and sorted by FADS [60,105,140], FACS [108,139] or AADS [94]. This strategy was used to screen mutants of the promiscuous *Pseudomonas aeruginosa* arylsulfatase for their capacity to transform a phosphonate molecule, a secondary substrate of the enzyme [60]. This led to the isolation of mutants with an overall increased expression level and a mutant six-fold more efficient. On another hand, the improvement of the detoxifying phosphotriesterase from *Pseudomonas diminuta* was of particular interest [108]. Indeed, whereas the µIVC procedure could identify mutants with a 19-fold improved activity toward the fluorogenic substrate analog used for the screening, the activity with the native substrate (the paraoxon pesticide) was increased only 8-fold, highlighting a potent limitation of using a fluorogenic substrate analog as the enzyme can evolve to recognize it better than the native substrate. Such a limitation can nevertheless be overcome by using the natural substrate of the enzyme and by detecting the reaction product with a chain of enzymatic reactions that convert the product into a fluorescent signal. This strategy was demonstrated for the detection of cellulase activity [143] as well as xylose consumption and lactate production [75]. Beside the natural enzymes, cell-based µIVC was also recently used to improve 30-fold the catalytic properties of an artificial computationally designed aldolase [140], opening new perspectives in the conception of artificial catalysts. Another µIVC strategy called DrOPS, recently allowed isolating a mutant of the 9n-GLK DNA polymerase (an engineered version of the DNA polymerase from *Thermococcus* sp. 9 °N) able to efficiently copy a DNA template into α-l-threofuranosyl xeno nucleic acid with a high degree of fidelity [139]. 

Whereas in most of the applications presented above the bacteria were lysed immediately upon their encapsulation, several works showed that integrating a picoinjection [73,74] or a fusion [75] step in the µIVC workflow further increases the flexibility of the process by successively performing steps with different timescale. This allowed, for instance, to first grow cells individualized into droplets for several hours (or days) prior to adding activity assay reagents and incubate the mixture for a few minutes before analyzing and sorting the droplets.

Mutational scanning, a strategy combining µIVC screening and next generation sequencing (NGS) represents an alternative strategy to improve enzymes in which the active mutants of a library are first sorted by µIVC before the sequences contained in both selected and unselected libraries are analyzed by NGS [105]. While this approach primarily allows for the discrimination of key residues (invariant) from those supporting variations, the application of a selection pressure and the insertion of cherry-picked mutations into the wild-type molecule allows the engineering of optimized enzymes, as demonstrated with the Bgl3 β-gluconidase from *Streptomyces* sp. whose thermostability was increased by more than 5 °C by a single point mutation identified by this approach.

All the screenings introduced above were performed with host cells expressing a library of mutant genes carried by a plasmid. However, another approach consists of introducing mutations at the level of the whole organism and selecting those survivals improved for the target activity. This was recently shown with the fungus *Aspergillus niger* and allowed the isolation of individuals displaying a significantly improved amylase activity among the rare survivals of a UV-irradiated population of spores [144].

Altogether, cell-based µIVC strategies are extremely attractive since they are cheap and they allow the isolation of catalysts not only improved for the target activity but also optimized in terms of expression level [75] and solubility [94], two important features when evolving molecules expected to be later produced at an industrial scale. Besides the direct improvement of the catalyst, cell-based µIVC can also be used to screen libraries of microbes prepared by UV irradiation and to search for host microorganisms with improved secretion properties [145,146].

### 5.2. Cell-Free Directed Evolution

µIVC-assisted directed evolution can also be performed in a cell-free manner using in vitro gene expression mixtures either made of purified components (in vitro transcription and Protein synthesis Using Recombinant Elements (PURE) [149] in vitro translation system) or based on cell extracts. This strategy ensures that all the droplets of an emulsion have identical composition, further increasing the accuracy of the method. In addition, using in vitro expression format allows for the expression of proteins toxic for a cell or even the introduction of non-natural amino acids. The first cell-free µIVCs were performed by mixing a cell extract-based in vitro expression mixture with a DNA solution on-chip just prior to producing the droplets [52,150]. Since DNA molecules distribute into the droplets following Poisson statistics [58,151], limiting dilution produces droplets containing at most one DNA molecule. The small volume of the droplets (femtoliters to picoliters) concentrates expression products to a detectable level [52,150,152], but working at the single molecule level may also lead to a significant variance resulting from gene-to-gene expression variation. Nevertheless, this variance can be mostly suppressed by increasing the copy number of gene molecules prior to expressing them [152]. Clonal population of a DNA molecule can easily be generated in droplets using isothermal [58] or PCR amplification [151,153]. However, since DNA amplification and gene expression are mutually exclusive events, a droplet fusion step is required upon DNA amplification to feed each droplet with the in vitro expression mixture [58]. Therefore, a typical cell-free µIVC screening of a mutant gene library occurs in three main steps: (i) DNA individualization and amplification, (ii) gene expression, and (iii) droplets analysis and sorting [104]. Whereas such a pipeline has not been applied to protein evolution yet, it has been used to improve the catalytic properties of RNA molecules [148] (Figure 7b), a process that would have been extremely challenging to perform using cell-based µIVC because of the strong cellular RNase background activity. Furthermore, the same procedure was used to improve the folding properties of the fluorogenic RNA aptamer Spinach by using selection pressures that would have been impossible to apply in a cell-based format (i.e., complete absence of potassium ions) [154]. Therefore, even though cell-free µIVC workflows are more complex than their cell-based counterparts, they are extremely valuable for applications requiring high control over reaction conditions and/or that are sensitive to cellular background.

### 5.3. Discovery of New Catalysts from Environmental Samples

Given the limited fraction of environmental microbes that are culturable [155], only a small fraction of the existing catalysts has been accessed so far, whilst the remaining microbiological dark matter has been explored mainly at a metagenomic level making possible only the identification of catalysts sharing homologies with the known enzymes [156]. Therefore, the ultrahigh-throughput regime of µIVC and its capacity to handle single cells make it an extremely attractive option for the functional screening of environmental samples during bioprospecting programs. This was recently demonstrated by the successful identification of cellulolytic microbes collected from a wheat stubble field [157]. Indeed, using a µIVC procedure, more than 100,000 microbes were screened in less than 20 min without the need of any pre-culturing step leading to the identification of several taxons with increased cellulobiohydrolase activity. Even more excitedly, another recent study used a cell-based µIVC approach to functionally screen a pool of metagenomic libraries from diverse sources cloned and expressed into *Escherichia coli* [147]. 1,250,000 variants were screened for promiscuous sulfatase and phosphotriesterase activities. Importantly, despite the fact that all these new enzymes shared a common activity, sequence-similarity networks showed that they did not strongly cluster together, but were distributed among various unrelated super-families. This finding is of great importance as it demonstrates that the sequence is insufficient for predicting a function and that functional screening by µIVC is a robust way to discover new catalysts. 

## 6. Conclusions and Outlook

In this review, we presented the main microfluidic modules that can be combined to devise µIVC screening procedures for performing highly quantitative measurements while giving great flexibility to the experimental workflows. The possibility of lysing cells on demand, either upon their encapsulation or after a defined culture time while preserving genotype/phenotype confinement has made this technology extremely attractive for cell-based screening performed at the single-cell level. While today only a handful of works has been reported, it is very likely that, in a near future, a plethora of new catalysts will be discovered and improved. The possibility of exploring the microbial dark matter should have a major impact on these new developments. The capacity of µIVC to identify the rare variants of interest contained in very large populations and at a very low cost makes a three-step scenario that could be routinely adopted in a near future highly plausible. In this scenario, µIVC would be first used to enrich environmental samples from various origins (e.g., agricultural soil, contaminated soils from industrial plant…) in organisms displaying the highest levels of the target activity (e.g., [157]). Then, metagenomic DNA libraries could be prepared from these microbes and a second µIVC screening could be used to identify the genes encoding for the target activity (e.g., [75,147]). Finally, the properties of the newly identified catalysts would be improved and tailored for their final application using one of the directed evolution workflows presented in this review. There is no doubt that the increasing number of companies selling microfluidic equipment and reagents will greatly boost new developments and offer a wider use of µIVC.
